# Hydride ions in Group II Metal (A = Ca, Sr, Ba) Titanate Oxyhydrides

**DOI:** 10.1063/4.0000845

**Published:** 2025-10-27

**Authors:** Kennedy Agyekum, Bernadette Cladek, Megan Burrill, Jue Liu, Katharine Page

**Affiliations:** 1 University of Tennessee, Knoxville, Knoxville, Tennessee, USA; 2 Northwestern University, Evanston, Illinois, USA; 3 Oak Ridge National Laboratory, Oak Ridge, Tennessee, USA

## Abstract

Perovskite oxyhydrides such as ATiO3-xHy (A = Sr, Ca, or Ba) offer flexible sublattices, high H- incorporation (up to 20 % of the anion site), and oxygen site vacancies to promote diffusion of both H- and O2-. Leveraging on both charge and mass transport, and the lability of H- at ≥ 400 ℃, this group of oxyhydrides is particularly useful for hydrogen storage, electrocatalysis, and energy conversion devices. However, our limited fundamental understanding of their defect chemistry and H- transport mechanisms hinder the progress of oxyhydrides in practical applications. Here, we have used neutron scattering and other complementary techniques to explore the local and average structure, quantify hydride content, and explore hydride transport kinetics in ATiO3-xHy, with a focus on Sr/CaTiO3-xHy. The neutron scattering results confirm successful H^−^ insertion, accompanied by increased cell volume and a high concentration of oxygen vacancies - both essential for fast ionic transport. From Rietveld refinement of our neutron diffraction data, we obtained two H levels in CaTiO3-xHy, resulting in CaTiO2.78H0.22 and CaTiO2.927H0.073.

For SrTiO3-xHy, we obtained SrTiO2.802H0.198 and SrTiO2.905H0.095 (CaH2-reduced SrTiO3), and SrTiO2.944H0.065 (NaBH4-reduced SrTiO3). Overall, CaH2-reduced samples contain higher levels of H- than NaBH4-reduced samples, and CaTiO3 takes up more H- than SrTiO3. With the same H- content, we are anticipating faster H- diffusion in the Ba and Sr versions of the solid solution series because of the polarizability of the Sr and Ba lattices relative to the Ca versions. The open question is whether H^−^ conductivity is primarily governed by H^−^ concentration, the crystal- chemical effects induced by particular A-site cations, or a combination of both. We plan to employ several complementary techniques to aid in structure determination.

